# Supplementary material for: Molecular mechanism and structural models of protein-mediated copper transfer to the Arabidopsis thaliana ethylene receptor ETR1 at the ER membrane
Source: Sci Rep. 2025 Nov 4;15:38501. doi: 10.1038/s41598-025-19915-6 (PMC12586476; doi:10.1038/s41598-025-19915-6)
Supplement: Supplementary file 1 — Supplementary Material 1 [file 41598_2025_19915_MOESM1_ESM.pdf]

Supplementary Information

Molecular Mechanism and Structural Models of Protein-Mediated Copper Transfer to the *Arabidopsis thaliana* Ethylene Receptor ETR1 at the ER Membrane

Dominik Dluhosch[1,4], Lisa Sophie Kersten[2,4], Alexander Minges[1], Stephan Schott-Verdugo[3], Holger Gohlke[2,3,5] and Georg Groth[1,5,*]

Affiliations

1 Institute of Biochemical Plant Physiology, Heinrich-Heine-Universität Düsseldorf, 40225 Düsseldorf, Germany.

2 Institute for Pharmaceutical and Medicinal Chemistry, Heinrich-Heine-Universität Düsseldorf, 40225 Düsseldorf, Germany.

3 Institute of Bio- and Geosciences (IBG-4: Bioinformatics), Forschungszentrum Jülich, 52425 Jülich, Germany.

4 Share first authorship

5 Share senior authorship

* Correspondence: Georg Groth (georg.groth@hhu.de)

Running title

Mechanism & Models for the Copper Transfer to ETR1

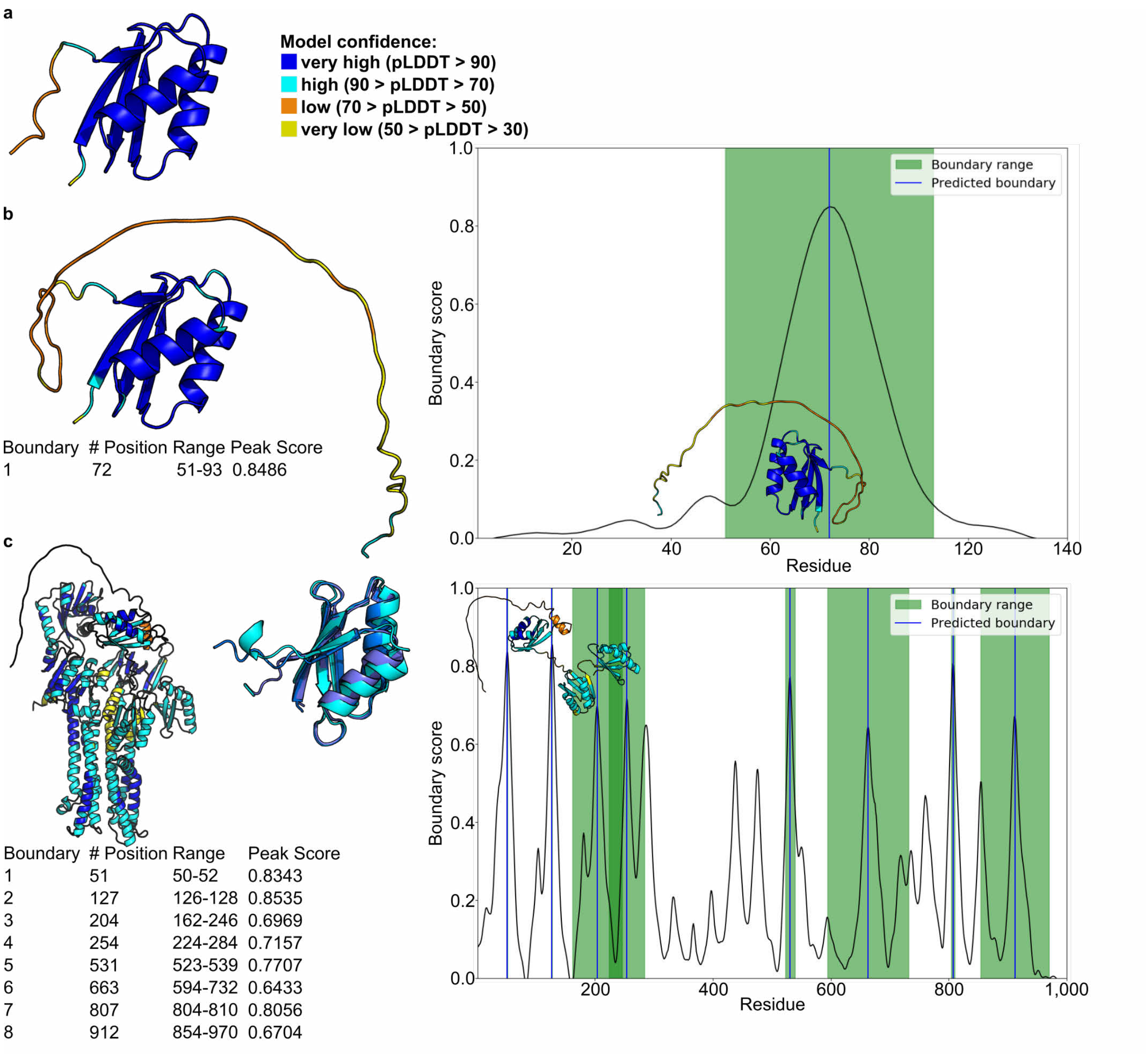

Supplementary Figure S1: Characterization of boundaries in CCH and RAN1 using AlphaFold2 and TopDomain. a: Representation of the structural model of ATX1 generated with AlphaFold. Colors correspond to the pLDDT, with dark blue indicating pLDDT > 90. The model has the ATX1-like fold. Residues in secondary structure elements have high pLDDT values. **b:** Representation of the structural model of CCH generated with AlphaFold. Colors correspond to the pLDDT. The model has the ATX1-like fold and residues located in secondary structure elements have high pLDDT values. The pLDDT values begin to fall from residue 69. TopDomain predicts a boundary between residues 51-93, with the highest boundary score at residues 52-93. For CCH to be comparable to ATX1, the boundary to the unstructured domain was set at residue 76. **c:** Representation of the structural model of RAN1 generated by AlphaFold. Colors correspond to the pLDDT. The structure has ATX1-like folds at the N-terminus. Sequence parts with these folds have pLDDT > 70 and are linked by loops with lower pLDDT values. The transmembrane domains and topological domains have pLDDT values of 70-90. TopDomain predicts boundaries between residues 50 to 53, 126 to 128, 162 to 246, and 224 to 284. Since residue 54 still has a pLDDT < 70, the beginning of MBD1 was set to 55. The end of MBD1 was set to 130 to include the ATX1-like fold and also some of the residues with lower pLDDT as in the case of ATX1 and CCH. MBD2 starts with the first residue involved in the second ATX1-like fold according to the AlphaFold model and ends with residue 207, to include additionally some of the residues featuring a lower pLDDT

as in the case of MBD1. MBD3 starts with residue 205, as the linker between MBD2 and MBD3 is only four residues long, and ends with residue 283 to include additionally some of the following residues.

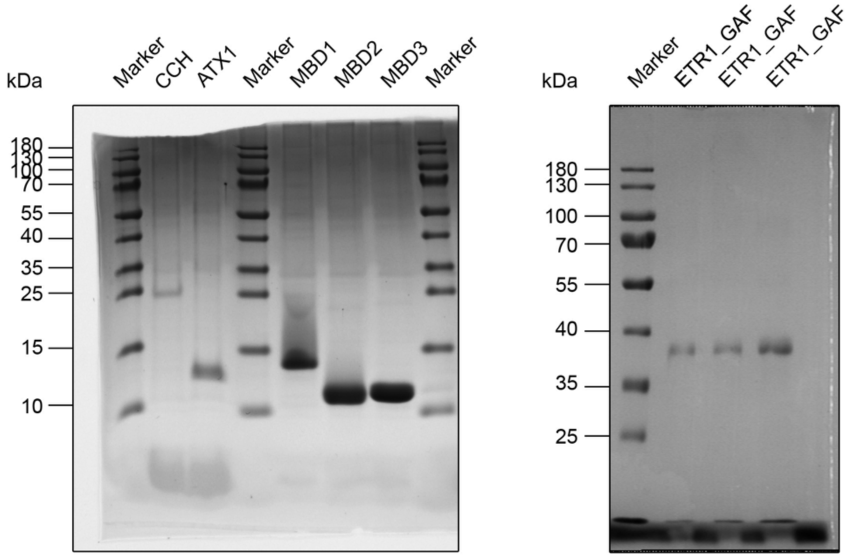

Supplementary Figure S2: Uncropped images of SDS-PAGE gels shown in Fig. 1. For ETR1_GAF, increasing amounts of protein were loaded onto the gel (1 µg, 2 µg and 3 µg, left to right) but only the lane containing 3 µg of protein and the marker lane are shown in Fig. 1. Gels were stained using Coomassie Brilliant Blue.

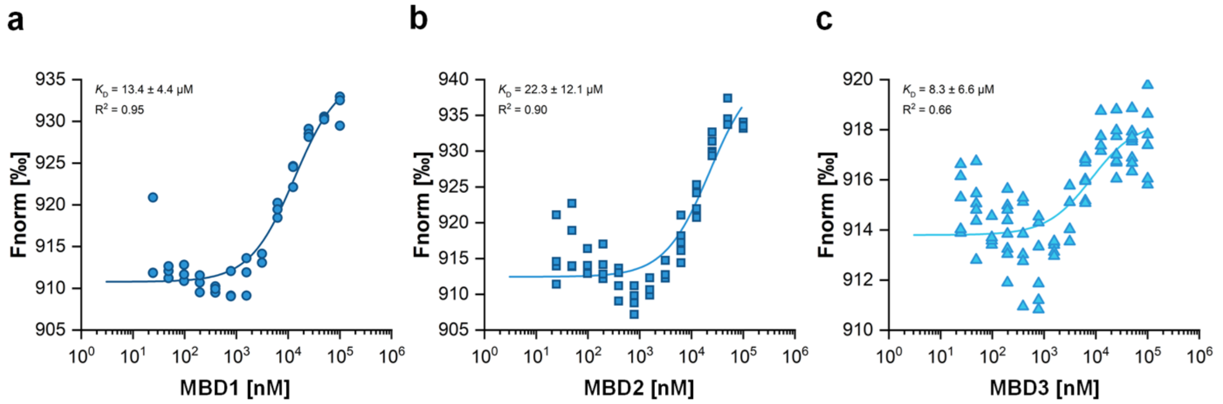

Supplementary Figure S3: Raw data underlying MST experiments on the interaction of ETR1_GAF with the individual RAN1 MBDs. In these experiments, ETR1_GAF is the labeled protein and is titrated with MBD1, MBD2 or MBD3. Lines represent fits to a one-site binding model.

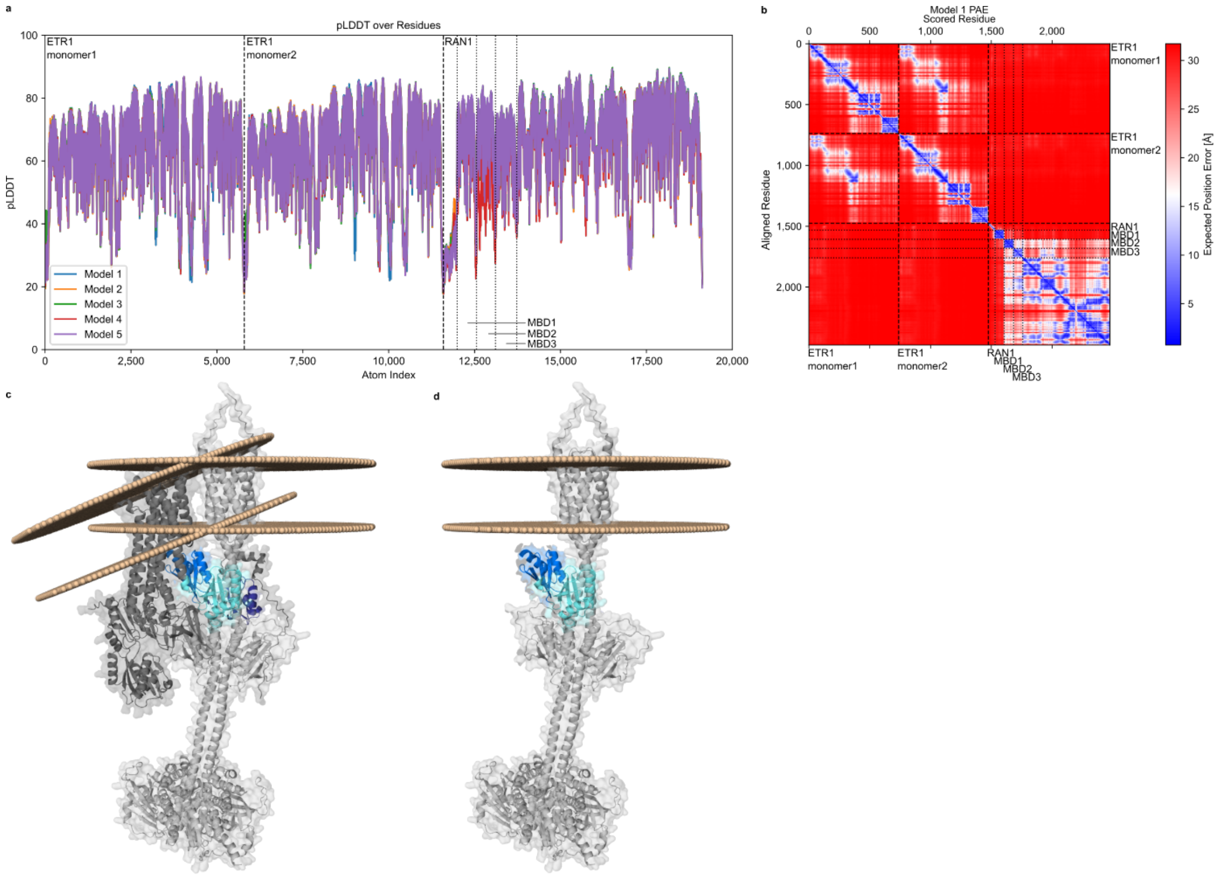

Supplementary Figure S4: RAN1/ETR1 complex predicted by AlphaFold3. Visual inspection suggests that models 1 to 3 are somewhat structurally related (RMSD < 10 Å) and similar to the complexes created by protein-protein docking (Figure 5). In rank1-3, all MBDs feature distances of less than 5 Å to ETR1, where MBD1 is located on the opposite side of ETR1 as MBD2 and 3. **a:** pLDDT of the corresponding models. Thick dotted lines indicate the boundaries of ETR1 monomer 1, ETR1 monomer 2, and RAN1, while thin dotted lines denote metal binding domain boundaries within RAN1. **b:** Predicted alignment error of model 1, also representative of models 2 and 3. Thick dotted lines indicate the boundaries of ETR1 monomer 1, ETR1 monomer 2, and RAN1, while thin dotted lines denote metal binding domain boundaries within RAN1. **c:** Membrane orientation of RAN1 and ETR1. The TMDs of RAN1 and ETR1 would not be located in the same membrane. **d:** Predicted binding positions of MBD2 and MBD3 of RAN1. MBD2 and MBD3 bind to the same domains as in the docking approaches. The remaining domains of RAN1 are hidden for the sake of clarity.

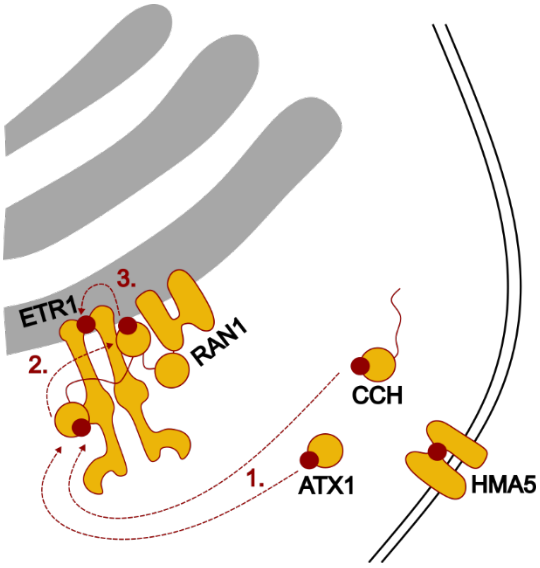

Supplementary Figure S5: Schematic representation of the copper relay hypothesis. MBD1 accepts the copper from the soluble copper chaperones ATX1 and CCH and transfers the copper to MBD2. Due to MBD2's proximity to the ETR1 TMD, MBD2 finally transfers the copper to ETR1. MBD3 acts as an anchor stabilizing the ETR1/RAN1 complex.

Supplementary Table S1: Data collection and refinement statistics. Statistics for the highest-resolution shell are shown in parentheses.

	MBD3 (PDB-ID: 8RNZ)
**Data collection**	
Wavelength (Å)	0.8856
Resolution range (Å)	51.48 – 1.98 (2.08 – 1.98)
Space group	$P2_12 2_1$
Unit cell	
a, b, c (Å)	50.755, 51.481, 62.722
α, β, γ (°)	90, 90, 90
Total reflections	80517 (11847)
Unique reflections	11943 (1670)
Multiplicity	6.7 (7.1)
Completeness (%)	99.80 (99.24)
Mean I/σ_I	5.81 (0.74)
Wilson B-factor	34.35
R_{merge}	0.1204 (0.5585)
R_{meas}	0.1306 (0.6044)
R_{pim}	0.04984 (0.2279)
$CC_{½}$	0.996 (0.836)
CC*	0.999 (0.954)
**Model and refinement**	
Reflections used in refinement	11941 (1668)
Reflections used for R_{free}	568 (69)
R_{work}	0.1922 (0.3236)
R_{free}	0.2349 (0.3587)
Number of non-hydrogen atoms	1309
Macromolecules	1229
Ligands	0
Solvent	80
RMSD	
Bond lengths (Å)	0.003
Bond angles (°)	0.540
Ramachandran analysis	
Favored regions (%)	100
Allowed Regions (%)	0
Outliers (%)	0
Rotamer outliers (%)	0
Clashscore	0.81
Average B-Factor	43.42
Macromolecules	43.34
Solvent	44.75

Supplementary Table S2: Secondary structure contents of MBDs 1 to 3 calculated from CD-spectra in the wavelength range from 190 nm to 250 nm. Secondary structure contents were estimated by the BeStSel webserver, with the secondary structure elements 3_{10}-helix, π-helix, bend, β-bridge, and loop/irregular regions being sorted into the category "others".

	**Secondary structure content [%]**				**Spectral deviation**
	**α-helical**	**β-sheet**	**turn**	**others**	**NRMSD**
MBD1	12.8	29.5	12.6	45.1	0.009
MBD2	8.8	32.4	13.6	45.2	0.010
MBD3	20.4	24.4	10.4	44.8	0.008

Supplementary Table S3: Primers used for cloning of the metal binding domains (MBDs) 1 to 3 of RAN1 from *Arabidopsis thaliana*.

Primer	Sequence [5´-3´]
**ATXfld-1-F**	*ctgtattttcagggattaaggaagattcaggtcgga*
**ATXfld-1-R**	*cggatcctcgagttattgtgtctgttcttcagcc*
**ATXfld-2-F**	*ctgtattttcagggaactttggtgggtcaatttac*
**ATXfld-2-R**	*cggatcctcgagttacttatcctgctgattactctg*
**ATXfld-3-F**	*ctgtattttcagggacagcaggataagcttgttttaag*
**ATXfld-3-R**	*cggatcctcgagttaataagggctcataacacgc*
**pETEV16b-F**	*taactcgaggatccggctg*
**pTEV-R**	*tccctgaaaatacaggttttcatgg*
